# Sources of variation in ancestral sequence reconstruction for HIV-1 envelope genes

**Published:** 2007-01-13

**Authors:** Howard A. Ross, David C. Nickle, Yi Liu, Laura Heath, Mark A. Jensen, Allen G. Rodrigo, James I. Mullins

**Affiliations:** 1 Bioinformatics Institute, University of Auckland, Private Bag 92019, Auckland, New Zealand;; 2 Department of Microbiology, University of Washington, Seattle, Washington 98195-8070, USA

**Keywords:** ancestral sequence reconstruction, center of tree, taxon sampling, rational vaccine design, HIV-1

## Abstract

We characterized the variation in the reconstructed ancestor of 118 HIV-1 envelope gene sequences arising from the methods used for (a) estimating and (b) rooting the phylogenetic tree, and (c) reconstructing the ancestor on that tree, from (d) the sequence format, and from (e) the number of input sequences. The method of rooting the tree was responsible for most of the sequence variation both among the reconstructed ancestral sequences and between the ancestral and observed sequences. Variation in predicted 3-D structural properties of the ancestors mirrored their sequence variation. The observed sequence consensus and ancestral sequences from center-rooted trees were most similar in all predicted attributes. Only for the predicted number of N-glycosylation sites was there a difference between MP and ML methods of reconstruction. Taxon sampling effects were observed only for outgroup-rooted trees, not center-rooted, reflecting the occurrence of several divergent basal sequences. Thus, for sequences exhibiting a radial phylogenetic tree, as does HIV-1, most of the variation in the estimated ancestor arises from the method of rooting the phylogenetic tree. Those investigating the ancestors of genes exhibiting such a radial tree should pay particular attention to alternate rooting methods in order to obtain a representative sample of ancestors.

## Introduction

Reconstructing proteins from ancestral genomic sequences (Cai et al 2004; Cunningham et al 1998) allows us to investigate the evolution of protein function (Chang and Donoghue 2000), or to construct biologically significant molecules. Many factors may affect the inference of ancestral sequences but assessment has tended to focus on the method of character state reconstruction on a given phylogenetic tree. Maximum likelihood (ML) had been shown to be more accurate than maximum parsimony (MP) (Koshi and Goldstein 1996; Thornton 2004; Zhang and Nei 1997) but there is recent evidence that optimality methods induce deterministic bias (Krishnan et al 2004).

There are several potential sources of error which could contribute to variation in the reconstructed ancestor. Generally we have no prior knowledge of which reconstructed ancestral sequence is correct, and so when investigating the properties of an ancestor, one might use several reconstruction methods to generate a population of potential ancestors which we think will capture method-induced variation (for example, Jermann et al 1995).

Deciding which methods to include in an experiment requires knowledge of the relative contributions of the methodological options to the variation in the reconstructed ancestor. Here we assess these sources of variation in ancestral sequence reconstruction, to assist in the choice of relevant methods to investigate in any particular application. Our focus is on software and methods which are readily available to investigators.

Ancestral sequence reconstruction is based on a phylogeny. We assess the variation arising from standard methods of estimating the phylogenetic tree. That is, do the different tree building methods create trees sufficiently different so as to have an impact on the reconstructed ancestor? We assess how the phylogenetic tree is rooted to define the ancestral node of the tree, whether by an outgroup or using a computed “center of tree”, an enhanced version of mid-point rooting that reduces the weight given to the most divergent taxa (Nickle et al 2003). We apply three computational approaches to inferring ancestral character states: MP (Edwards and Cavalli-Sforza 1963; Farris 1970; Swofford and Maddison 1987), ML (Yang et al 1995; Zhang and Nei 1997) and empirical Bayes’ (Huelsenbeck and Bollback 2001; Schultz et al 1996). Lastly, we assess the variation due to the informational level (nucleotide, codon or amino acid) at which the reconstruction is performed.

The biological properties of predicted ancestors are best screened first by computational methods. Only later would it be economic to synthesize and analyze some of the postulated proteins from the reconstructed sequences (Chang and Donoghue 2000). By using structural modeling and expert systems, we compare the variation in predicted characteristics of the hypothesized ancestral proteins with the variation in predictions obtained for the observed sequences.

Little is known about the effects of taxon sampling, ie the number and selection of ingroup sequences, on ancestral sequence reconstruction. A logarithmic improvement in ancestral state reconstruction using MP has been demonstrated with increasing taxon number (Salisbury and Kim 2001) and a relatively modest number of taxa are required to have reasonable confidence that a phylogenetic tree includes the true root of the clade (Sanderson 1996). Here we used a random deletion method to determine empirically the effect of taxon sampling.

**Figure 1:**
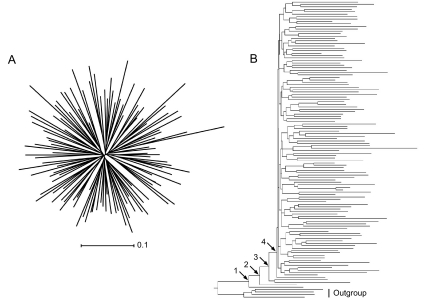
An NJ phylogenetic tree of the HIV-1 *env* sequences used in this study: (A) unrooted and (B) rooted using an outgroup. See the text for a discussion of nodes 1, 2, 3 and 4.

This assessment was conducted in the context of Human Immunodeficiency Virus type 1 (HIV-1) vaccine design, an area of vigorous study (Mullins et al 2004), but our results will be of interest to investigators of other biomolecules. Between-host variants of HIV-1 usually form phylogenetic trees with a star topology, with long terminal branches and short branches near the root, as a result of rapid divergence (Figure 1A). It has been hypothesized that a suitable vaccine might be designed using a reconstructed ancestral protein sequence, because it will represent a sequence most similar to all circulating strains and may therefore elicit a stronger immunologic response than other candidate vaccines (Gaschen et al 2002; Learn and Mullins 2000; Mullins et al 2004; Nickle et al 2003). We identify the placement of the root and selection of the out-group as potentially significant sources of variation in the ancestral sequence as it relates to HIV-1 vaccine design (Gaschen et al 2002; Nickle et al 2003; Novitsky et al 2002). Our purpose here is not to present a vaccine candidate but to identify those methodological stages that contribute to the variation in reconstructed ancestral sequences, and hence potential vaccines, These results will also assist investigators of other such systems in designing their computational experiments and in deciding where to direct expensive laboratory resources.

## Methods and Materials

### Observed Sequences

The primary data comprised 118 B-subtype DNA sequences from the C2-V5 region of the HIV-1 envelope glycoprotein (*env*) gene (GenBank accession numbers AY139268 - AY139370, AY139372, AY139374 – AY139381, AY139383 – AY139388), each obtained, one per subject, by the U.S. Centers for Disease Control and Prevention in five different cities in the United States (Anderson et al 2001). As outgroups, we selected four D-subtype sequences at random (GenBank accession numbers K03454, M27323, U88822, U88824), and the consensus sequences for the M group and the D subtype, all from the LANL HIV-1 sequence database (Korber et al 2003). The D subtype is the sister group of the B subtype within the M group of HIV-1 (Korber et al 2000).

### Experimental Design and Coding

**Figure 2:**
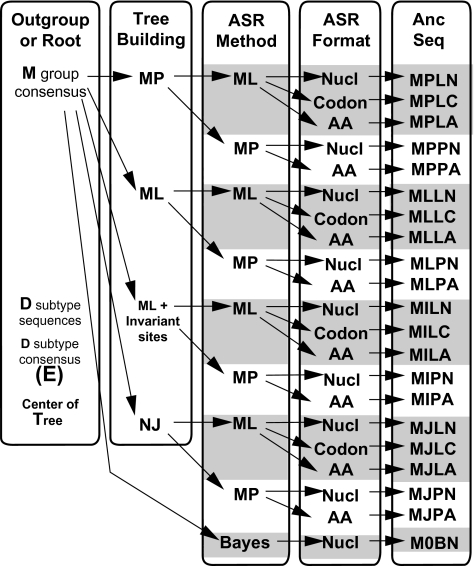
Each ancestral sequence reconstruction was given a 4-letter code in which each position indicates sequentially (a) how the ancestral node was defined, (b) the method of tree building, (c) the method of ancestor reconstruction and (d) the format in which the ancestor was reconstructed. All potential reconstructions for a single outgroup are shown, while a similar number were considered for the other outgroups and methods of rooting the tree.

The ancestral sequence for the observed sequences was derived using combinations of different methods of tree estimation and sequence reconstruction (Figure 2 and below), except where they have not been implemented in software or are meaningless. Some combinations were abandoned when computation time exceeded 2 months.

### Alignments

A DNA alignment of the observed sequences was obtained with ClustalX v1.81 (Thompson et al 1997), and then adjusted manually to restore the reading frame and the translated amino acid sequence reported in GenBank. The consensus of the aligned DNA sequences was then recorded (**Con**). Three additional DNA alignments included the M-group consensus, the D-subtype consensus or the D-subtype sequences, again preserving the translation.

### Rooting the tree

Phylogenetic trees were estimated for each of the four alignments. Three sets of trees were rooted using as outgroup the four D-subtype sequences (**D**-rooted), the D-subtype consensus (**E**-rooted) or the M-group consensus (**M**-rooted). The fourth phylogenetic tree, lacking an outgroup, was rooted at the center (**T**-rooted), estimated as the point in the tree having the least squared distance to the branch tips (Nickle et al 2003).

### Tree Estimation

The phylogenetic tree was estimated by: (1) maximum parsimony (MP) using PAUP* v4.0b10 (Swofford 2002) and a heuristic search with the starting tree obtained by stepwise addition and branch swapping using the TBR algorithm (**P**-tree); (2) neighbor joining (NJ) using PAUP*, with distance estimated using maximum likelihood under the GTR+Γ model (**J**-tree); (3) maximum likelihood (ML) using PAUP*, starting with the NJ tree, and then using maximum likelihood under the GTR+Γ model and TBR branch swapping (**L**-tree); (4) ML with a fixed proportion of invariant sites using PAUP*, where the fixed proportion of invariant sites (pI = 0.1254) had been estimated on an ML **D**-rooted tree (**I**-tree); while (5) in the empirical Bayes’ analysis, no tree was estimated (**0**-tree).

### Ancestral Sequence Reconstruction

The ancestral sequence was reconstructed at the node basal to the clade of observed sequences as: amino acids (**A**-format), codons (**C**-format) and nucleotides (**N**-format).

1. The **B**-method used Bayesian phylogenetic inference and Markov chain Monte Carlo (MCMC) using MRBAYES v2 (Huelsenbeck and Ronquist 2001). No phylogenetic tree was estimated (**0**-tree), but the ancestral node was specified by constraining the sequence reconstruction to the node basal to the clade containing the observed sequences. The outgroup sequences were excluded from this constrained group, effectively rooting the tree (**D**-rooted, **E**-rooted or **M**-rooted). Only **N**-output ancestral sequences were reconstructed. The results report the sequence obtained from a single chain of 3 × 10^7^ generations, under the GTR+Γ+I model, but see the online Supplementary Materials for further discussion.
2. ML reconstructions (**L**-method) were obtained using PAML v3.13 (Yang 1997). **A**-format sequences were reconstructed using *codeml*, an amino acid alignment, and Jones substitution matrix (Jones et al 1992). **C**-format sequences were reconstructed using *codeml*, a DNA alignment, empirical codon frequencies, empirical nucleotide frequencies estimated separately by codon position, one estimate of the nonsynonymous: synonymous substitution ratio ω across all branches, and one estimate of the transition: transversion ratio κ. **N**-format sequences were reconstructed using *baseml*, a DNA alignment, GTR model of nucleotide substitution with one rate across all branches, empirical nucleotide frequencies, one estimate of the transition: transversion ratio κ. In each case a marginal reconstruction was performed and the rate heterogeneity among sites was modeled by the Γ-distribution with α estimated using 4 bins.
3. MP reconstructions (**P**-method) were obtained using PAUP*, to give **A**-format or **N**-format sequences.

### Comparisons among reconstructed ancestral sequences

When comparing reconstructed sequences we deleted unreliable sites where gaps in the observed sequences had a frequency of 0.50 or greater. These gaps arose through alignment with outgroup sequences or observed sequences with rare insertions.

### Structural Prediction

The variation in the tertiary structure of ancestral sequences was assessed using (a) threading (reported in the online Supplementary Material) and (b) the variation in the energy of the structure predicted by the spatial constraints method (Sali and Blundell 1993). A few protein sequences were first submitted to the Swiss-Model comparative modeling protein server (http://www.expasy.org/swissmod/) to identify suitable structural templates for subsequent modeling. The three templates identified (PDB IDs: 1g9mG, 1g9nG, and 1gc1G) are each derived from HIV-1 B-subtype sequences with the V3 loop mutationally excised. The observed and ancestral sequences were aligned with the template sequences, again pruned both of unreliable sites and of sites that did not occur in the templates, and then modeled using Modeller (Sali and Blundell 1993) to obtain the energy of the inferred structure. Again, an energy with a larger negative value indicates a better fit to the templates.

### Immunological Prediction

All of the original and ancestral sequences were searched for potential epitopes. Again, sites where gaps had a frequency of 0.50 or greater were deleted. Then ungapped sequences were compared with all of the antibody (Ab), helper T-cell (HTL) and cytotoxic T-cell (CTL) epitopes recorded in the LANL HIV-1 immunology database (Korber et al 2003).

Further, the number of CTL epitopes was predicted using MHCPred (Guan et al 2003), which predicts the 9-mer sites recognized by a MHC Class I allele, for all of the alleles supported by MHCPred in late 2003 (A*0101, A*0201, A*0202, A*0203, A*0206, A*0301, A*1101, A*6801, A*6802, and B*3501). The sites were ranked by their predicted affinity threshold (IC_50_), the concentration (nM) for which 50% binding is predicted. Sites were categorized as having high (IC_50_ ≤ 50 nM), intermediate (IC_50_ in range 50–500 nM) or low (IC_50_ in range 500–5000 nM) affinity (Sette et al 1994). Analysis was limited for practical reasons to ancestral sequences reconstructed from **D-** and **T-**rooted trees, representing the two types of tree rooting, and all observed sequences. Reconstructed ancestral sequences were submitted unaltered, and MHCPred ignored any ambiguity code in the sequence.

### N-glycosylation Prediction

The number of acceptor sites for N-linked glycosylation on each sequence was estimated using NetNGlyc (Gupta et al 2002) with the default prediction threshold.

### Taxon Sampling

The full set of B-subtype sequences was subsampled by removing a proportion of sequences selected at random. The proportions of sequence removed were 10% (i.e. leaving 90% intact), 25%, 50%, 75% and 90%, with 100 replicates of each. For each replicate sample, the phylogenetic tree was estimated using NJ (**J**-tree as above). The trees were rooted either by using the D-subtype sequences as an outgroup or at the center (**D**- and **T**-rooted trees). The rooted tree was also generated by pruning the selected taxa from the full tree based on all sequences. Then, the ancestral nucleotide sequence was predicted both by ML and by MP (**L**-method and **P**-method as above). The variation in the reconstructed ancestral nucleotide sequence was assessed by computing the proportional difference (*p*-distance) from the appropriate reference ancestral sequence reconstructed using all 118 B-subtype sequences. There were four reference sequences, each determined using a combination of the method of rooting the tree (outgroup vs center) and the method of reconstructing the ancestral sequence (ML vs MP).

## Results

### Sequence variation among reconstructed ancestral sequences

**Figure 3:**
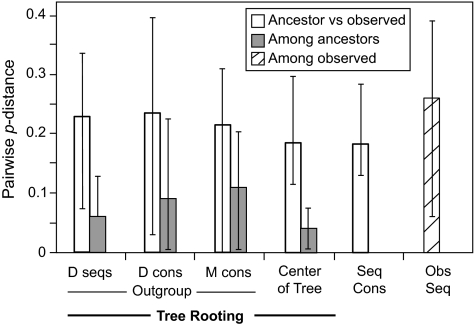
Genetic distances between observed and ancestral sequences. The mean (error bars indicate range) pairwise *p-*distance between each ancestral sequence and the observed sequences (white bars), within each group of ancestral sequences (grey bars), and among the observed sequences (hatched bar), for ancestral sequences from phylogenetic trees rooted with an outgroup: D-subtype sequences (**D**), D-subtype consensus (**E**), M-group consensus (**M**), or at the Center of Tree (**T**), and the consensus of the observed sequences (**Con**).

**Figure 4:**
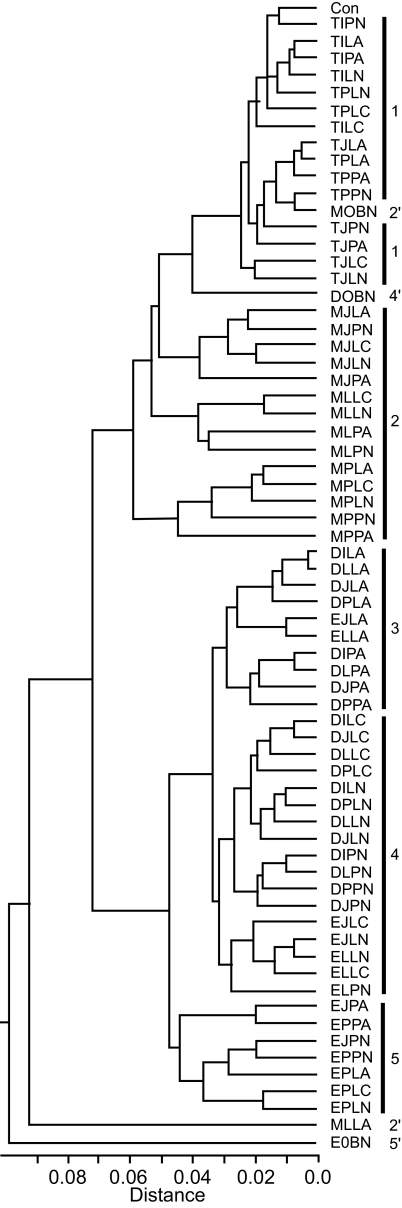
UPGMA tree constructed from pairwise *p*-distances among ancestral sequences expressed as amino acids. Each ancestral sequence is coded by the method by which it was derived (Figure 2). **Con** is the consensus of the observed sequences. Vertical bars delimit groups based on method of rooting the phylogenetic tree. The sequences labeled 2′, 3′, 4′ and 5′ indicate outliers related to groups 2 to 5 respectively.

Ancestral sequences reconstructed under many combinations of methods (Figure 2) were compared as amino acid sequences. On average the pairwise *p*-distance between ancestral sequences (mean: 0.11, range: 0.005 – 0.23) was only half that among the observed sequences (mean: 0.26, range: 0.06 – 0.39) (Figure 3). When ancestral sequences were grouped by the method of rooting the phylogenetic tree, mean *p-*distance was least among sequences reconstructed at the center of the tree, increased through sequences reconstructed using D-subtype sequences or consensus as the outgroup, and was greatest when the M-group consensus was used as the outgroup. Clustering the sequences by UPGMA (Swofford 2002) identified major groups based on the method used to root the phylogenetic tree (Figure 4). The first cluster (group 1) contained all of the ancestral sequences from the center of the tree and the consensus of the observed sequences. Otherwise this cluster had little structure with respect to the other reconstruction techniques used here. The next cluster (group 2) comprised ancestral sequences derived using the M-group consensus as the outgroup, within which the sequences clustered according to the method used in phylogenetic tree construction. The second half of the tree included reconstructions which arose when the D-subtype sequences or consensus were the outgroup, in three subgroups: one (group 3) contained cases where the ancestor was reconstructed as amino acid sequences, another (group 4) contained ancestral sequences reconstructed as codon or nucleotide sequences, and the third (group 5) contained a miscellany of reconstructions. Four outlier sequences, including the three sequences reconstructed using Bayesian methods, fell distant from other sequences reconstructed with the same rooting method. Similar results were obtained from multidimensional scaling, and are available online as Supplementary Material.

### Sequence differences between reconstructed ancestral and observed sequences

**Figure 5:**
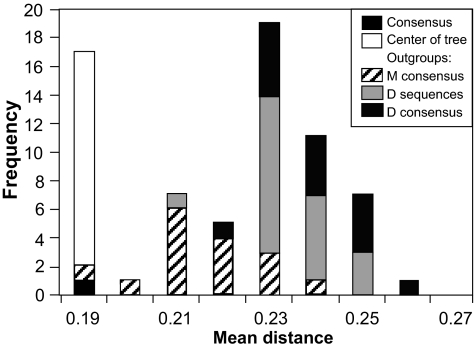
Frequency distribution of the mean *p*-distance between an ancestral sequence and each of the observed sequences separated by the method of rooting the phylogenetic tree.

While there was considerable variation in the extent to which the ancestral sequences differed from observed sequences, from being identical to being different at one-third of amino acid sites (Figure 3), the major differences were primarily among the methods of rooting the phylogenetic tree, and only secondarily between the methods of reconstructing the ancestral sequence on those trees. The distribution of mean *p-*distance between an ancestral sequence and the original sequences was multimodal (Figure 5). The greatest similarity with observed sequences was found for the set comprising the consensus of the observed sequences, the ancestral sequences reconstructed at the center of the tree (group 1 in Figure 4) and an ancestral sequence reconstructed using the Bayesian method (outlier 2′ in Figure 4). When the M-group consensus was used as the outgroup, the reconstructed ancestral sequences (group 2′ in Figure 4) had a higher mean and greater range in distance values. Among this group of sequences, those reconstructed by MP tended to have greater mean distances to the observed sequences than did those reconstructed using ML. The highest mean distances arose when the D-subtype sequences or consensus were used as the outgroup (groups 3, 4 and 5 in Figure 4). Among this set of ancestral sequences, those reconstructed by MP were significantly more distant from the observed sequences than were those reconstructed by ML (Mann-Whitney U test, *P* < 0.001).

### Effect of the outgroup on the reconstructed ancestral sequence

The outgroup has two potential effects on variation in the reconstructed ancestors, indirectly in the placement of the root node on the tree, and directly through its influence on the reconstruction of character states at interior nodes in the tree. For some experimental situations, the ancestral sequence was reconstructed a second time on a rooted tree while excluding the actual outgroup sequence from the alignment. The relative magnitudes of the outgroup effects were assessed by considering the difference between paired sequences, reconstructed with or without the outgroup sequence, or with different positions of rooting. The presence of the outgroup sequence (D-subtype consensus vs M-group consensus) was responsible for a 10% difference in the ancestral sequence while the position of the root node (as set by the outgroups or at the center of the tree) was responsible for a 14% difference. By comparison, the method of inferring the ancestral sequence on the tree (e.g. ML vs MP) generated a difference of 9%, regardless of whether the out-group sequence was present. More details are given in the Supplementary Materials.

### Variation in predicted structural properties

**Figure 6:**
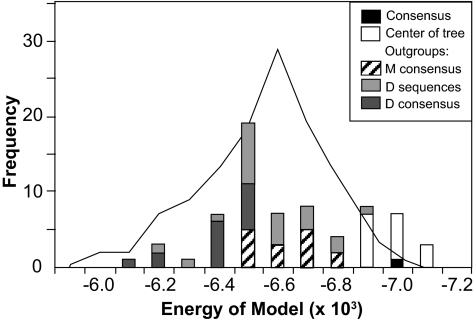
Energy of the 3-D structure for each observed and ancestral sequence predicted using the Modeller software and 3 structural templates. The frequency distribution of model energy is plotted for the observed sequences (line), and the consensus and ancestral sequences (bars) separated by the method of rooting the phylogenetic tree. Energies with larger negative value indicate better model fit.

The distribution of model energies predicted by Modeller for the observed sequences was symmetrical, but over-dispersed, while for the ancestral sequences the distribution was bimodal (Figure 6). A lower negative value for model energy indicates a poorer fit of the predicted structure on the template structures. Lower values were observed for ancestral sequences reconstructed on trees rooted with an outgroup, among which significantly lower energies were observed when MP, rather than ML, was used to reconstruct the ancestor (Mann-Whitney U test, *P* < 0.001). In contrast, higher model energies were found for the consensus of the observed sequences and for ancestral sequences reconstructed on trees rooted at the center. However there was no discernible pattern of variation due to the method of reconstruction among these sequences. Similar differences between ancestral sequences reconstructed on outgroup-rooted trees and on center-rooted trees were observed when threading was used to identify the protein fold (see online Supplementary Materials).

### Variation in predicted number of epitopes

**Figure 7:**
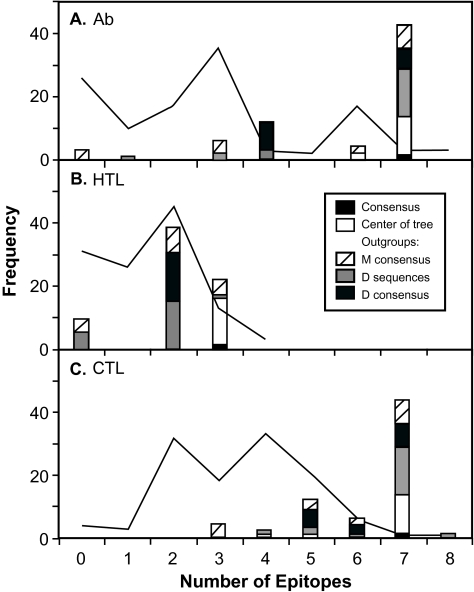
Frequency distributions of the number of (**A**) Ab, (**B**) HTL and (**C**) CTL epitopes identified by database searching for the observed sequences (line), and the consensus and the ancestral sequences (bars) separated by the method of rooting the phylogenetic tree.

Potential Ab, HTL and CTL epitopes, identified by sequence comparison with epitope databases, were found in most of the observed and ancestral sequences (Figure 7). In general, the ancestral sequences had very similar epitope profiles, containing all but the rarest epitopes in the observed sequences. Of the epitopes with frequency > 5% in the observed sequences, the majority were common (frequency > 50%) in the ancestral sequences (7 of 8 Ab, 2 of 4 HTL, and 7 of 8 CTL epitopes). No rare epitopes (frequency < 5% in the observed sequences), nor additional epitopes, were observed in the ancestral sequences. Single uncommon HTL and CTL epitopes (frequency 5–6% among observed sequences) were reconstructed only under Bayesian analysis, in the case when the D-subtype sequences were used to constrain the ingroup, but not when either the D-subtype or M-group consensus sequences formed the outgroup. The average number of epitopes observed in each of the ancestral sequences was greater than in the observed sequences (Table 1). Ancestral sequences reconstructed on trees rooted at the center had the greatest mean and smallest range in number of epitopes, while the lowest mean and greatest range in number of epitopes were found when the M-group consensus formed the outgroup. The consensus of the observed sequences had the greatest number of each type of epitope.

### Variation in predicted MHC binding sites

The number and binding affinity of T-cell epitopes or recognition sites, predicted by MHCPred to occur on each sequence, did not show clear differences on the basis of reconstruction method. In general, the majority of predicted binding sites had low predicted binding affinity, while sites with medium binding affinity were intermediate in number, and high binding affinity sites least common. The ancestral and observed sequences were compared by jointly ranking them on the number of predicted binding sites. The higher ranks among all sequences were dominated by those for ancestral sequences reconstructed using likelihood, either as codons or nucleotides, or from ancestral sequences reconstructed on trees rooted using the D-subtype sequences as the outgroup, depending on the way in which the average ranks were computed. The consensus of the observed sequences had an intermediate score in all aspects of this analysis. However, the number of predicted binding sites was not supported by the number of epitopes inferred by comparing sequences against databases of known epitopes (Table 1). The results of the analysis are given in greater detail in the online Supplementary Materials.

### Variation in predicted number of N-glycosylation binding sites

**Figure 8:**
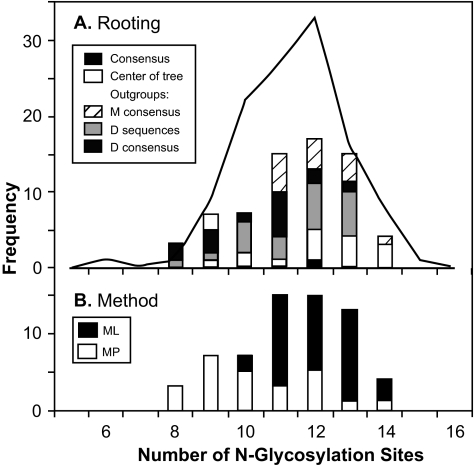
Frequency distribution of the number of predicted N-glycosylation sites in each observed or ancestral sequence: (**A**) the frequency distribution plotted for the observed sequences (line), and the consensus or the ancestral sequences (bars) separated by the method of rooting the phylogenetic tree; (**B**) the same ancestral sequences, excluding the consensus, separated by the method of reconstructing the ancestral sequence.

The number of N-glycosylation binding sites predicted by NetNGlyc to occur in each sequence varied among both observed and ancestral sequences (Figure 8). The number of binding sites in the ancestral sequences varied in a similar manner to the number in the observed sequences, and there was no pattern discernible with respect to the way in which the phylogenetic tree had been rooted (Figure 8A). However, sequences reconstructed by ML had a significantly greater number of binding sites than did sequences reconstructed by MP (Mann-Whitney U test, *P* < 0.001).

### Taxon Sampling

**Figure 9:**
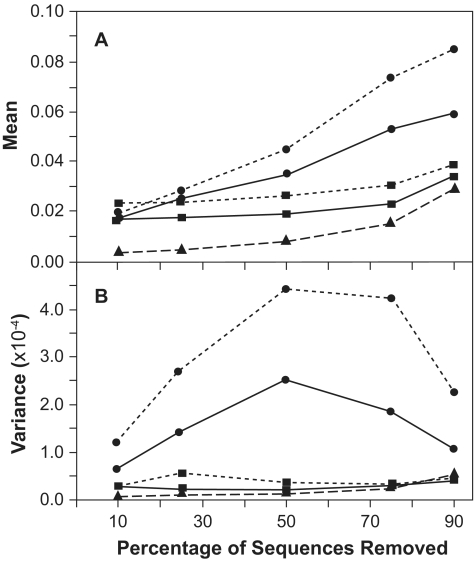
(A) Mean and (B) variance (N = 100) of the divergence of ancestral sequences reconstructed from a subset of sequences relative to that reconstructed for the entire sample of 118 sequences. circles - rooted using outgroup (D-subtype sequences); squares - rooted at center of tree; dotted line - MP reconstruction; solid line - ML reconstruction; triangle/dashed line - consensus of subsample.

Overall, the ancestral sequence reconstructed from a subsample became progressively more divergent from the reference sequence as the size of the subsample decreased (Figure 9). The pattern of divergence differed qualitatively according to how the tree was rooted, and quantitatively according to the method used to reconstruct the ancestral sequence.

When the tree was rooted using D-subtype sequences as the outgroup, the mean divergence of ancestral sequences increased relatively linearly with increasingly smaller subsamples (Figure 9A) whereas the variance in divergence was lower when either a large or small number of sequences was used, and was greater at intermediate numbers of sequences (Figure 9B). At low percentages of sequence removal, sequences reconstructed via MP or ML were equally similar to the reference ancestral sequences, but as more sequences were removed, sequences reconstructed using MP became more divergent than sequences reconstructed using ML, and showed greater variance.

In sharp contrast to ancestral sequences reconstructed on trees rooted with an outgroup, the mean and variance in divergence of sequences reconstructed at the center of the tree remained low across most of the range of sample sizes (Figure 9A). Only when 90% of the sample was removed did the mean divergence increase. The mean divergence of sequences reconstructed by MP was slightly greater than when ML was used. The change in the divergence of the consensus of the sequences in the subsample was similar in form, but of lower magnitude, to that for sequences reconstructed at the center of the tree.

The pattern of divergence observed for ancestral sequences reconstructed on trees rooted with the outgroup might have arisen because the placement of the root changed on the phylogenetic tree having fewer leaves. To test the effect of altered rooting, the original phylogenetic tree of 118 taxa was pruned of those taxa chosen for deletion, thereby leaving the root as close to its original position as possible, and the ancestral sequence was reconstructed. In all 10 cases involving the outgroup (5 proportions deleted X 2 methods), the mean divergence of the ancestral sequences on the pruned tree was less than on the re-estimated tree (results not shown) whereas for trees rooted at the center, differences in mean divergence were observed only when small proportions of sequences were deleted. These observations suggested that the position of the root was changing significantly on outgroup-rooted trees, but not on center-rooted trees, as the number of sequences was reduced. However, by examining the individual pairs of trees (re-estimated and pruned), we found that the position of the root for outgroup-rooted trees had changed only at the higher percentages removed (10%: 0; 25%: 2; 50%: 13; 75%: 63; 90%: 82, each of 100 cases). In contrast, for center-rooted trees the position of the root had changed in every one of 500 cases. Clearly a confounding factor was involved.

**Figure 10:**
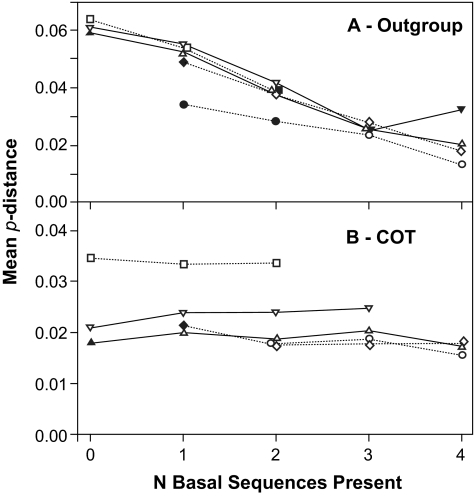
Mean divergence of ancestral sequences reconstructed using ML on subsets of sequences from that reconstructed for the entire sample of 118 sequences in relation to the number of basal sequences present (i.e. those joining the rooted tree at nodes 1, 2, and 3 in Figure 1) for ancestors estimated on phylogenetic trees (A) rooted using an outgroup and (B) rooted at the center. Symbols: circle - 10% removed; diamond - 25% removed; triangle - 50% removed; inverted triangle - 75% removed; square - 90% removed; filled - very small sample sizes (N ≤ 5).

Closer inspection of the whole tree rooted using an outgroup (Figure 1B) revealed four sequences on relatively long branches near the base of the ingroup. It was hypothesized that the presence of these sequences in the sample had a significant effect on the placement of the root, and consequently on the ancestral sequence. When some or all of these sequences were present, the ancestral sequence was reconstructed at nodes 1, 2 or 3, but when all were absent then node 4 became the ancestral node. The replicate subsamples were separated on the basis of the number of these basal sequences present and the mean and variance of divergence were recalculated. For sequences reconstructed on outgroup-rooted trees there was an inverse relationship between the mean divergence and the number of these basal sequences present, for both ML (Figure 10A) and MP (similar results, not shown). Furthermore, the variance in divergence for each size of subsample was positively correlated with the variance in the number of these basal sequences present in the subsample (Spearman r_S_ = 0.9), for either method. In contrast, for sequences reconstructed at the center of the tree we found that these basal sequence had no direct effect on the reconstructed ancestral sequence (Figure 10B) for either method of reconstruction. Divergence was constant with respect to the number of the basal sequences present in the subsample, and changed only on the basis of the size of that subsample.

## Discussion

The variation in the sequence and structural characteristics of the ancestral gene sequences reconstructed by different methods was dominated by the effect of the method used to root the phylogenetic tree on which the reconstructions were based. The method used to reconstruct the ancestral sequence, or the type of the reconstructed sequence, had only secondary relevance to the variation observed. Generally the technique used to estimate the phylogenetic tree was irrelevant to the outcome. For only one of the immunological characters estimated, the number of N-glycosylation sites, were ancestral gene sequences strongly influenced by the analytic method used to reconstruct the ancestral sequence.

Previous assessments of ancestral state reconstructions have focused on the accuracy of the method, rather than the variation among methods. Although parsimony has been shown to reconstruct true ancestors with a high degree of accuracy in laboratory settings involving the serial propagation of bacteriophage (Bull et al 1993; Hillis et al 1992), likelihood methods have been considered to be more accurate (Huelsenbeck and Crandall 1997; Koshi and Goldstein 1996; Thornton 2004; Yang et al 1995; Zhang and Nei 1997) because of more realistic model specification. Empirical Bayes’ methods (Huelsenbeck and Bollback 2001; Schultz et al 1996) extended these optimality methods by integrating across tree topologies to choose the most likely character state. Krishnan and colleagues (2004) have recently demonstrated however that each of these optimality methods introduces a deterministic bias in nucleotide frequencies and that the entire posterior probability distribution of ancestral states should be considered when inferring ancestral function. However, they take the phylogenetic tree as a given, including the taxa represented, the tree’s topology and its root, and do not investigate the variation arising from these factors. Pagel and colleagues (2004) assessed confidence in the state reconstructed at internal nodes of the tree, including the root, by assessing variation across trees sampled by MCMC and in particular by considering the occurrence of the node. When a node exists in only some of the potential phylogenetic trees, but the reconstruction is constrained to those trees which contain that node (for example, Huelsenbeck and Bollback 2001), Pagel and colleagues showed that confidence in the reconstruction is over-estimated.

The study reported here was initiated prior to the publication of Krishnan’s and Pagel’s results and used methods and software tools which are commonly available to investigators. While the optimality methods used in all reconstructions may introduce bias, we may ask how important that bias is in the presence of the other factors investigated. Because the set of input sequences was constant, each reconstruction was conceptually of the same ancestor, and so we expected the variation in the ancestor to be less than that of the input sequences. However, the range of values of the structural and immunological attributes predicted computationally for the inferred ancestral sequences matched that of the input sequences (Figures 6, 7, 8). Both when the variation was due primarily to the method of rooting the tree, as for the predicted structure, or when it was due to the method of reconstruction, as for the number of predicted N-glycosylation sites, the range of values associated with each technique (eg, MP or ML) was a significant proportion of the range of values predicted for the input sequences. While optimality methods of sequence reconstruction may induce deterministic biases in inferred properties (Krishnan et al 2004), it appears that the other factors in the current study either compensate for, or overwhelm, the magnitude of these biases.

It is perhaps perplexing that the rooting method should have such significance when previous studies have revealed major differences between MP and ML. This may be due in part to the strongly radial form of the phylogenetic tree (Figure 1A). Rooting with one outgroup or another will often cause the ancestral node to be placed differently on the tree, in either case somewhere other than at the center. However the actual sequence of the outgroup also introduces variation through its influence on the reconstruction of character states at interior nodes of the tree. This outgroup effect may simply be a common source of variation which is routinely overlooked in assessments of reconstruction methods. The magnitude of this outgroup effect should relate to the divergence of the out-group sequence from the most recent common ancestor with the ingroup. With tree topologies having a less radial topology, as when branch lengths are sampled by a Yule process, the placement of the root should be less sensitive to the choice of outgroup. However, as we show here, the outgroup affects both the placement of the root and the reconstruction of character states at internal nodes, so that an appreciable outgroup effect is still to be expected.

Whereas ML and MP are well-established techniques for ancestral reconstruction, Bayesian methods have not had wide use. Here, the empirical Bayes’ method reconstructed ancestors very different from other ancestral sequences reconstructed on a rooted tree, and from one another, in three cases using different sets of sequences as the constraining outgroup (Figure 4), and in other analyses reported in the online Supplementary Material. Because the phylogenetic tree for HIV-1 has short internal branches and long terminal branches (Figure 1), our data may lack sufficient phylogenetic information for reliable determination of character states at nodes near the root. Under the empirical Bayes’ method, the ancestral state for each site is the nucleotide having the greatest posterior probability given the outgroup constraints and the phylogenetic trees visited during MCMC sampling. So at highly variable sites the nucleotide selected may have only a modest posterior probability (*P*), approaching one-quarter in the limit. While most sites had nucleotide assignments with very high posterior probability (*P* ≫ 0.99), 5–9% of sites had *P* < 0.9 and 2–3% of sites had *P* < 0.7. Our results suggest that empirical Bayes’ methods may in some situations, especially those with larger data sets, contribute to variation in the reconstructed ancestor. Methodological improvements may help to reduce that variation (Krishnan et al 2004).

The center-of-tree approach to mid-point rooting of the phylogenetic tree minimizes the effect of highly divergent taxa on the tree (Nickle et al 2003). Given the relatively symmetric and starlike phylogenetic tree for HIV-1 (Figure 1A), it is unsurprising that the observed sequence consensus shared many attributes with ancestral sequences estimated using center-of-tree rooting. Ancestors estimated using outgroups must be more divergent than the consensus or those obtained from a center-of-tree root because these outgroups are more distant (Korber et al 2000, their Fig. 1).

While we have studied the variation in ancestral sequences reconstructed on star-like phylogenetic trees (Figure 1), we recognize that for other tree topologies the factors associated with tree and sequence estimation may make different relative contributions to the variation in the reconstructed ancestor. Nevertheless, we have restricted ourselves to this one topology because, in our experience, highly infectious viruses such as HIV-1 and influenza exhibit such topologies and because this study was initiated in the context of identifying potential vaccine candidates. We wished to understand the variation in the ancestor which might arise from the methods used. An examination of ancestral reconstruction on other topologies, while desirable, is beyond the scope of this study.

Taxon sampling, that is whether the sample is representative of natural sequence variation, may be a source of variation in the reconstructed ancestor. Our data set is based on viral extracts from infected individuals sampled in a short time in many widespread US cities (Anderson et al 2001), and thus reduces the potential issue of having some clades over- or under-represented by recent clonal expansion within a host or host population. Here the contribution of taxon sampling to the variation in the reconstructed ancestor lay not so much in the number, as in the nature, of the input sequences, especially when the phylogenetic tree was rooted with an outgroup (Figure 9). A small number of relatively divergent sequences falling near the root played a dominant role in specifying the ancestor (Figures 1B, 10). Although sequences reconstructed on outgroup-rooted trees became progressively more different, and highly variable, at smaller taxon samples, this trend was due to the frequency of occurrence of these divergent sequences. In contrast, ancestors reconstructed on trees rooted at the center were largely unaffected by taxon sampling. These divergent sequences did not fall near the root when it was placed at the center of the tree, and consequently had less significance.

We might conjecture that the variation in ancestral sequences reconstructed on trees rooted at the center will always be lower. Because the tree center is defined as the point having the least squared distance to the leaves, the proportionate effect of a few long branches at the edge of the tree will be much less than when an outgroup is used to root the tree. Also, the standard method for midpoint rooting, using the midpoint of the longest path on the tree, should be more sensitive to the inclusion of highly-divergent taxa in the sample.

The impact of these basal taxa on outgroup-rooted trees will have significance for the sampling strategy of studies designed to characterize ancestral sequences. When speciation and extinction occur at constant rates over time, Sanderson (1996) has shown that only a relatively modest sample size is needed for one to be 95% confident that the phylogenetic tree includes the basal node for a large clade. Our results suggest that recombination or introgression may be an unexpected and greater source of variation in the reconstructed ancestor. For example, in viruses such as HIV-1 (McCutchan et al 1999) or FIV (Bachmann et al 1997; Casado et al 2001), putative recombinant sequences usually fall on a phylogenetic tree between the hypothesized parental strains or subtypes. In such cases the inclusion of recombinant sequences in the sample may have a significant impact on the ancestor reconstructed. Similarly systematic bias in the sampling of natural sequence variation may result in greater variance in the estimated basal node, and ancestral sequence.

The HIV-1 envelope glycoprotein elicits a strong humoral response from the host, and is consequently under strong selection for sequence change in order to evade epitope recognition by neutralizing antibodies, cytotoxic T lymphocytes and helper T lymphocytes (McMichael and Phillips 1997; Phillips et al 1991; Ross and Rodrigo 2002) and to acquire surface-bound carbohydrates to mask the protein from host surveillance (Reitter et al 1998; Wei et al 2003). In the context of HIV-1 vaccine design, our results suggest that the method of rooting the phylogenetic tree and the method of reconstructing the ancestor contribute to variation in predicted immunological properties, and so should be investigated when reconstructing ancestral sequences in order to obtain promising candidate vaccine sequences. On the other hand, the methods of tree construction, and the format in which the sequence was reconstructed, made only slight contributions to variation in the ancestral sequence. So, computationally efficient methods, such as using neighbor-joining to estimate the phylogenetic tree and using nucleotides as the information level, may be adequate to sample suitable candidate vaccines.

Studies of the biochemical evolution of ancient biomolecules or their application to biotechnology must cope with uncertainty in the estimation of the ancestral sequence. One approach to assessing this uncertainty is to use Bayesian methods to estimate the posterior probability distribution of the ancestor. Another approach, used here, is to use different methods to obtain a sample of potential ancestors. Our results clearly show that, in the case of a radial phylogeny, variation in the sequence and structural properties of the ancestors arose from variation in the method of rooting the phylogenetic tree. When pragmatic decisions must be made regarding resource allocation in the design of an experiment to investigate possible ancestors more computationally intensive methods of tree or sequence reconstruction may not be necessary when simpler methods will give an adequate representation of the ancestor-space.

## Supplementary Materials

### 1. Multidimensional Scaling of Differences among Ancestral Sequences

**Figure S1:**
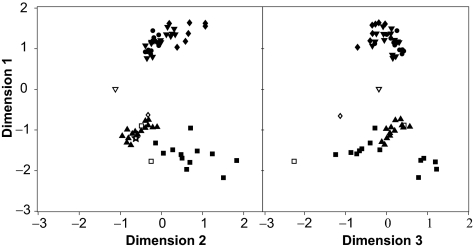
Multidimensional scaling of the *p-*distances among reconstructed ancestral sequences. The ancestral sequences are: T-rooted sequences [group 1 (triangles)], **M**-rooted sequences [group 2 (squares)], **D**- and **E**-rooted sequences [groups 3 (circles), 4 (inverted triangles) and 5 (diamonds)], and the consensus (**Con**) of the observed sequences (star).

Multidimensional scaling (Young 1996) was used to summarize in 3 dimensions the distance relationships among the reconstructed ancestral sequences, to determine whether they clustered according to some combination of the techniques used in reconstruction (Figure S1). The clustering among the ancestral sequences was similar to that found using UPGMA. Dimension 1 (40.4% of the variation in pairwise distance) effectively segregated **T**- and **M**-rooted sequences [groups 1 (triangles) and 2 (squares)] from **D**- and **E**-rooted sequences [groups 3 (circles), 4 (inverted triangles) and 5 (diamonds)]. Dimension 2 (8.5% of the variation) separated group 1 from group 2, and groups 3 and 4 from group 5. Dimension 3 (7.2% of the variation) differentiated the subgroups of group 2 (**M**-rooted) with the outlier **MLLA** on the far left, **P**-tree sequences on the far right, and **L**- and **J**-tree ancestral sequences in the middle. The **B**-method sequences were persistent outliers with respect to the method of tree rooting. The consensus of the observed sequences (star), expressed as amino acids (Con), was embedded in group 1. Open symbols identify the outliers observed in both UPGMA and MDS analyses.

### 2. Effect of Outgroup Sequence

When the phylogenetic tree is rooted using an outgroup, there are two potential sources of variation in the reconstructed ancestors: the indirect effect of the placement of the root node on the tree, and the direct influence of the outgroup sequence on the reconstruction of character states at interior nodes in the tree. The relative magnitudes of these two effects were investigated by reconstructing the ancestral sequence on rooted trees, but with the outgroup sequence removed from the input sequence alignment. These reconstructed ancestral sequences were then compared with ancestral sequences reconstructed with the outgroup sequence present. This analysis was performed for phylogenetic trees rooted using either the D-subtype consensus (**E**-rooted) or the M-group consensus (**M**-rooted) sequences as the outgroup, with the tree inferred by maximum likelihood (**L**-tree), and the sequences reconstructed using either maximum likelihood (**L**-method) or maximum parsimony (**P**-method). These combinations include experimental codes **MLL**–, **MLP**–, **ELL**–, and **ELP**–. Ancestral sequences reconstructed at the center of the tree, where the tree was estimated using ML with invariant sites in the model (experimental codes **TIL**– and **TIP**–), were included in the comparison because these reconstructions necessarily lack an outgroup sequence and differ from the others primarily in the placement of the root node.

**Figure S2:**
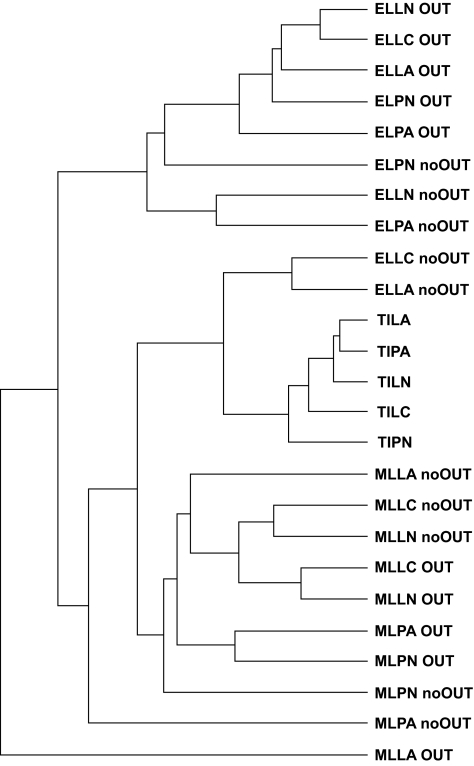
UPGMA clustering of ancestral sequences reconstructed when the outgroup sequence was present (OUT) or absent (noOUT), while retaining the position of the root node.

The reconstructed ancestral sequences segregate under clustering into groups primarily on the basis of the rooting method (Figure S2). Within those groups involving an outgroup, there is a tendency for sequences reconstructed when the outgroup sequence was present to segregate from those reconstructed when the outgroup sequence was absent. Clustering on the basis of the method of inference of the ancestral sequence is less apparent.

To assess the two effects of outgroup rooting, the root node placement and the outgroup sequence itself, the proportional difference between paired sequences was determined (Table S1). The presence of the outgroup sequence was responsible for a 10% difference in the ancestral sequence while the position of the root node was responsible for a 14% difference. By comparison, the method of inferring the ancestral sequence on the tree generated a difference of 9%, regardless of whether the outgroup sequence was present. Sample sizes in these comparisons are small so statistical significance of these differences cannot be determined. Nevertheless, it is apparent that both effects of outgroup rooting make an important contribution to variation on the reconstructed ancestral sequence.

### 3. Prediction of Structural Properties

The variation in tertiary structure of the ancestral sequences was assessed using threading to predict the protein fold. Amino acid sequences were submitted to GenTHREADER (McGuffin et al 2003), which returned a predicted fold and a neural network-derived measure of confidence in the prediction (Jones 1999; McGuffin and Jones 2003). The fold prediction included the most similar protein structure in the Protein Data Bank (Berman et al 2000), the pairwise potential of mean force between the atoms in the proposed structure of the submitted sequence and those of the reference structure (*E_pair_*), and the mean solvation potential for the amino acid residues in the proposed structure (*E_solv_*). *E_pair_* measures the similarity of the 3-D structure of the query protein to that of the model. *E_solv_* measures the degree to which amino acid residues are buried within the 3-D structure, as opposed to being accessible to the solvent molecules. Predicted energies with larger negative values indicate more favorable structures or better fit to the template. In preliminary investigations, the HIV-1 Gp120 Core protein (PDB ID1gc1G0) was the only reference structure returned. This protein structure lacks the variable region loops [amino acid residues 298–324 and 394–403 in the B-subtype consensus (Korber et al 2000)] which had been mutationally excised prior to structure determination (Kwong et al 1998). The sequences subsequently submitted to GenTHREADER were pruned both of sites where gaps occurred (see above) and of sites that were not in the 1gc1G0 reference. Analysis was limited for practical reasons to ancestral sequences reconstructed from **D-** and **T-**rooted trees, representing the two ways of tree rooting, and all observed sequences.

**Figure S3:**
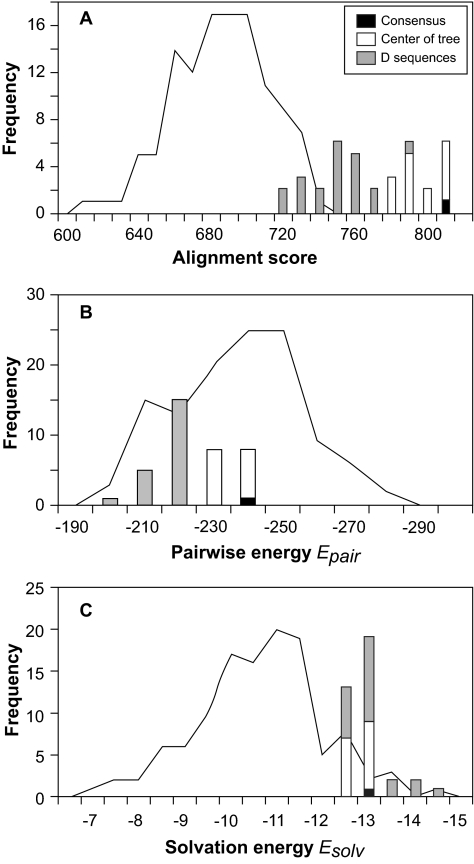
Frequencies distributions for measures of the quality of threading predictions of the protein fold for the observed (line) and ancestral sequences (bars), reconstructed from **D**- or **T**-rooted phylogenetic trees: (**A**) the sequence alignment score (greater alignment score indicates greater sequence similarity to inferred fold), (**B**) the pairwise energy (greater negative score indicates better model fit) and (**C**) the solvation energy (greater negative score indicates a more favorable structure in an aqueous environment).

GenTHREADER identified the same protein fold (PDB ID1gc1G0) with high confidence (E-value = 0.002) for every observed and ancestral sequence. The distribution of alignment scores reported by GenTHREADER for the original sequences was approximately normal in shape and only slightly overlapped that for the ancestral sequences (Figure S3A). The major source of variation was the method of rooting the phylogenetic tree with **D**-rooted sequences having lower alignment scores than **T-**rooted sequences and the consensus of the original sequences (**Con**). A lesser source of variation was the method of reconstruction, when only among **D-**rooted sequences did **P**-method sequences have lower scores than **L**-method sequences. A small amount of the difference may be attributable to the slightly longer sequence alignments (166 residues) obtained for the ancestral sequences than for the observed sequences (161–165 residues).

The distribution of pairwise energy potential (*E_pair_*), a measure of the predicted fit of the query sequence to the reference protein fold, was approximately normal for the observed sequences, but skewed for the ancestral sequences, with nearly all ancestral sequences having values below the mean value for the observed sequences (Figure S3B). A lower negative value of *E_pair_* indicates a poorer fit of the sequence onto the reference fold. Again the major source of variation was the tree rooting method, with the *E_pair_* scores for **D**-rooted sequences lower than for **T**-rooted sequences and the consensus of the original sequences (**Con**). Again a lesser source of variation was the method of reconstruction, when only among **D**-rooted sequences, **P-**method sequences had lower scores than **L**-method sequences.

The distribution of the energy of solvation (*E_solv_*), a measure of the energy potential of the molecule in a solvent environment, was strongly modal for the observed sequences, and skewed for the ancestral sequences, all of which fell in the upper quartile of the distribution for the observes sequences (Figure S3C). Again, a lower negative value indicates a less favorable state. The ancestral sequences had little variation in *E_solv_^,^* and no methodological differences distinguished those with a high score.

The skew in the distributions of the alignment scores, *E_pair_* and *E_solv_* for the ancestral sequences may be attributable to the fact that the various reconstructed ancestors are essentially all estimates of the same entity, and do not constitute a random sample from the space of all sequences. Nevertheless, for two of the three parameters the method of rooting the phylogenetic tree was the greatest source of variation in the reconstructed ancestor.

### 4. Prediction of MHC Binding Sites

**Figure S4:**
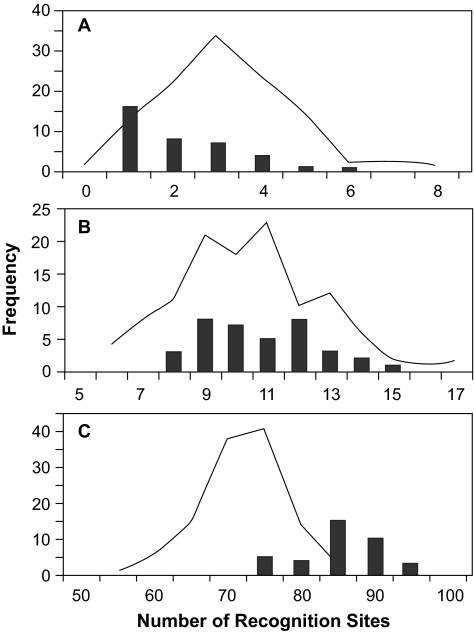
Frequency distributions of the number of HLA binding epitopes predicted by MHCPred to occur in each observed sequence (line) or ancestral sequence reconstructed from phylogenetic trees using either D or T rootings (bars). These three graphs illustrate the range of variation observed: (**A**) sites with predicted high binding affinity to allele HLA A*0203, (**B**) sites with predicted medium binding affinity to allele HLA A*0301, and (**C**) sites with predicted low binding affinity to allele HLA A*1101.

The number and binding affinity of T-cell epitopes or recognition sites, predicted by MHCPred (Guan et al 2003) to occur on each sequence, varied considerably both among sequences and among MHC Class I alleles. The observed sequences and **T**- and **D**-rooted ancestral sequences, representing the two types of rooting the phylogenetic tree, were analyzed. Figure S4 illustrates the variation in number of binding sites, showing cases where the distribution for the reconstructed ancestral sequences (A) fell below, (B) was coincident with, or (C) was above the distribution for the observed sequences. The scales on the figure axes also illustrate that the number of predicted sites formed the series low > medium > high binding affinity, although the magnitude of the range varied among alleles.

To assess whether the number of predicted recognition sites varied with the parameters being examined, the observed and reconstructed ancestral sequences were ranked jointly with respect to the number of predicted sites, for each allele and binding affinity. These ranks were then averaged across alleles. See the next section for the average rankings.

High affinity recognition sites were relatively rare, and for only four of the 10 alleles were two or more such sites predicted for any sequence, either B-subtype or reconstructed ancestral. **D**-rooted ancestral sequences had higher average ranks than did **T**-rooted sequences. Overall, reconstructed ancestral sequences were widely distributed throughout the ranks of all sequences.

Medium affinity recognition sites were relatively common, with at least one predicted in every sequence for seven of the 10 alleles, and in the great majority of sequences for nine of the 10 alleles. **T**-rooted ancestral sequences had higher rankings than **D**-rooted sequences, among which the lowest average ranks were for **P**-method sequences. Again, reconstructed ancestral sequences were widely distributed throughout the ranks of all sequences.

Low affinity recognition sites were very common, and were predicted to occur on every sequence investigated. The highest average ranks were dominated by **D**-rooted, **L**-method sequences. Almost all of the reconstructed sequences had higher average ranks than did the observed sequences, with only a few **T**-rooted sequences falling among the observed sequences.

Taking the gross average of rank across levels of binding affinity, we find a predominance of **L**-method and **C**- or **N**-format ancestral sequences at high average rank. If a weighted average is taken using logarithm-scaled weights (high 1.0, medium 0.1, low 0.01) then the highest overall ranks are from **D-**rooted sequences. The consensus of the observed sequences achieved an intermediate score in all aspects of this analysis.

The number of epitopes predicted by MHCPred can be compared with the frequency of known CTL epitopes in the **D**- and **T**-rooted ancestral sequences and the consensus of the observed sequences (Table 1 in paper). Whereas the consensus sequence was predicted to have only an intermediate ranking in terms of the number of epitopes, in fact it contained the same number (i.e., 7) as did the majority of **D**-rooted (16/21) and **T**-rooted (13/15) ancestral sequences. The prediction that **D**-rooted sequences should have more epitopes was not supported by epitope database search (**D**-rooted: 16/21 vs **T**-rooted: 13/15), and the prediction that **L**-method sequences should be superior to **P**-method sequences was only weakly supported (19/21 vs 10/14).

### 5. Average Relative Rank in number of sites recognised by MHC Class I alleles as predicted by MHCPred

Observed sequences have ID of form AY****** and reconstructed ancestral sequences have 4-letter ID code indicating method of reconstruction.

Ancestral sequences have been color-coded by method of tree rooting and format of sequence reconstruction.


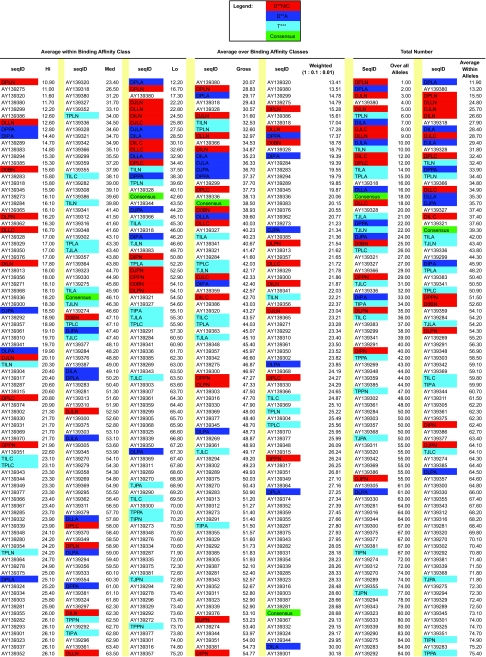
 
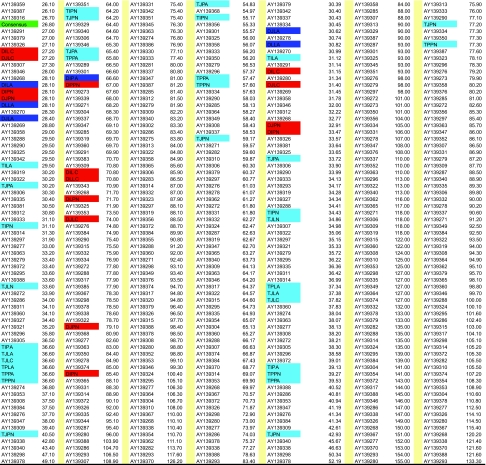


### 6. Inference of Ancestral Sequences by Bayesian Methods

To assess the variability in the ancestral sequences reconstructed using empirical Bayes’ methods, the reconstruction obtained using the D-subtype consensus as the constraining outgroup (**E**-rooted) was repeated for different numbers of generations or with more chains.

The following settings were used:

- 10 million generations, sampled at intervals of 500 generations on 1 or 4 chains to give 20,000 samples (e0bn10M1 and e0bn10M4).
- 30 million generations, sampled at intervals of 500 generations, on 1 chain to give 60,000 samples, with 2 replicates (e0bn30Ma and e0bn30Mb). One of these replicates is the same as E0BN, as reported in the main document.
- 60 million generations, sampled at intervals of 1000 generations, on 1 chain to give 60,000 samples with 2 replicates (e0bn60Ma and e0bn60Mb).

**Figure S5:**
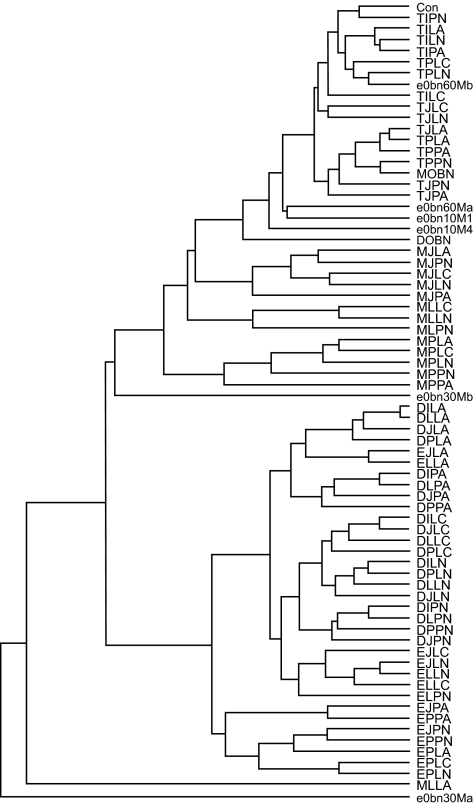
UPGMA clustering of the reconstructed ancestral sequences including 6 reconstructions performed using Bayesian methods and the D-subtype consensus as the constraining outgroup (**E**-rooted). See the text for a description of the computational conditions for the Bayesian reconstructions.

**Figure S6:**
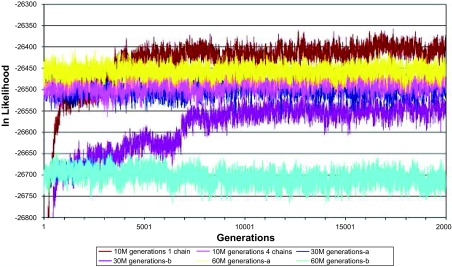
The likelihood of each sampled tree from the 6 Bayesian reconstructions of the ancestor, computed using the D-subtype consensus as the constraining outgroup (**E**-rooted). Only the last 20,000 samples are shown in each run. For the runs involving 30 million or 60 million generations, this chart shows the last third whereas for the shorter runs of 10 million generations this chart shows the entire run.

The replicate ancestral sequence reconstructions are widely divergent (Figure S5). Figure S6 plots the likelihoods of the sampled trees from the 6 MCMC analyses listed above. Only the last 20,000 samples are plotted for each run, which for the shortest analyses represents the entire run while for the longer analyses it represents the last third. Each trace shows a relatively stable trace, after the initial phase in the runs involving 10M generations, but there is considerable difference among the runs. The run using 4 chains achieves the highest likelihood. However when we compare the likelihood achieved and the position of the ancestral sequence in the cluster analysis, we see that there is no relationship between sequence similarity and likelihood. Sampling statistics calculated for these 6 analyses (Table S2) indicate that each analysis superficially had a reasonably good effective sample size. However, the failure of the analyses to show convergence suggests that lack of mixing is a significant issue. For trees having a radial topology, as used in this study, it may be that Bayesian analyses, or at least the implementation in MrBayes v2, is not appropriate for reconstructing ancestral sequences, and that the difficulty of demonstrating that a convergent solution has been achieved presents a serious operational issue.

**Table S1:** Mean (SD) *p*-distance between paired ancestral sequences, reconstructed with a) outgroup sequence present or absent, b) different positions of the root node, and c) different methods of sequence inference (ML vs MP).

a) Outgroup sequence present or absent				
Outgroup	D-subtype (**E**)	M-group (**M**)		Combined
Mean (SD)	0.09 (0.03)	0.10 (0.06)		0.10 (0.04)

b) Different positions of the root node				
outgroup sequence present:				
Outgroup	**E** vs **M**			Combined
Mean (SD)	0.17 (0.02)			0.17 (0.02)

Outgroup sequence absent:				
Outgroup	**E** vs **M**	**E** vs **T**	**M** vs **T**	Combined
Mean (SD)	0.18 (0.04)	0.13 (0.06)	0.12 (0.03)	0.14 (0.05)

c) Method of inference (ML vs MP)				
Outgroup sequence present				
Outgroup	D-subtype (**E**)	M-group (**M**)		Combined
Mean (SD)	0.05	0.14		0.09 (0.07)

Outgroup sequence absent:				
Outgroup	D-subtype (**E**)	M-group (**M**)	Center of Tree (**T**)	Combined
Mean (SD)	0.10	0.12	0.03	0.09 (0.04)

**Table S2:** Sampling statistics for the 6 Bayesian reconstructions of the ancestor, computed using the D-subtype consensus as the constraining outgroup (**E**-rooted). Burnin = initial set of samples discarded before computation of tau (IACT = integrated Autocorrelation time) and ESS (Effective Sample Size, i.e. the effective number of “independent” samples).

Generations	Burnin	Tau	ESS
10M, 1 chain	2k	230	78
10M, 4 chains	2k	118	153
30M, replicate 1	10k	176	284
30M, replicate 2	10k	324	154
60M, replicate 1	2k	127	456
60M, replicate 2	2k	333	174

## Figures and Tables

**Table 1:** Immunologic epitopes predicted for reconstructed sequences. The number of neutralizing antibody (Ab), helper T-cell (HTL) and cytotoxic T-cell (CTL) epitopes identified in each observed sequence, their consensus, and in each ancestral sequence, are reported. The first three columns are the mean number of each type of epitope per sequence, while the last two columns are the mean and standard deviation (SD) of the total number of all of these epitopes per sequence.

Sequences	Ab	HTL	CTL	All (Mean)	All (SD)
B-subtype					
Observed sequences	2.8	1.4	3.4	7.6	3.0
Sample Consensus	7	3	7	17	-
Ancestral sequences					
**D**-rooted	5.9	1.6	6.7	14.1	2.8
**E**-rooted	5.2	2.0	6.0	13.2	2.1
**M**-rooted	4.4	1.7	5.4	11.5	4.7
**T**-rooted	6.9	3.0	6.7	16.5	1.2
